# 1,25-Dihydroxyvitamin D(3) Inhibits Podocyte uPAR Expression and Reduces Proteinuria

**DOI:** 10.1371/journal.pone.0064912

**Published:** 2013-05-31

**Authors:** Jianchao Ma, Bin Zhang, Shuangxin Liu, Shaoting Xie, Yun Yang, Juan Ma, Yujun Deng, Wenjian Wang, Lixia Xu, Ruizhao Li, Li Zhang, Chunping Yu, Wei Shi

**Affiliations:** 1 Southern Medical University, Guangzhou, China; 2 Department of Nephrology, Guangdong General Hospital, Guangdong Academy of Medical Sciences, Guangzhou, China; 3 Department of Emergency Medicine, Guangdong General Hospital, Guangdong Academy of Medical Sciences, Guangzhou, China; Fondazione IRCCS Ospedale Maggiore Policlinico & Fondazione D’Amico per la Ricerca sulle Malattie Renali, Italy

## Abstract

**Background:**

Accumulating studies have demonstrated that 1,25-Dihydroxyvitamin D(3) (1,25(OH)2D3) reduces proteinuria and protects podocytes from injury. Recently, urokinase receptor (uPAR) and its soluble form have been shown to cause podocyte injury and focal segmental glomerulosclerosis (FSGS). Here, our findings showed that 1,25(OH)2D3 did inhibit podocyte uPAR expression and attenuate proteinuria and podocyte injury.

**Methodology/Principal Findings:**

In this study, the antiproteinuric effect of 1,25(OH)2D3 was examined in the lipopolysaccharide mice model of transient proteinuria (LPS mice) and in the 5/6 nephrectomy rat FSGS model(NTX rats). uPAR protein expression were tested by flow cytometry, immune cytochemistry and western blot analysis, and uPAR mRNA expression by real-time quantitative PCR in cultured podocytes and kidney glomeruli isolated from mice and rats. Podocyte motility was observed by transwell migration assay and wound healing assay. Podocyte foot processes effacement was identified by transmission electron microscopy. We found that 1,25(OH)2D3 inhibited podocyte uPAR mRNA and protein synthesis in LPS-treated podocytes, LPS mice and NTX rats, along with 1,25(OH)2D3 reducing proteinuria in NTX rats and LPS mice.1,25(OH)2D3 reduced glomerulosclerosis in NTX rats and alleviated podocyte foot processes effacement in LPS mice. Transwell migration assay and wound healing assay showed that LPS-induced podocyte motility, irrespective of random or directed motility, were substantially reduced by 1,25(OH)2D3.

**Conclusions/Significance:**

Our results demonstrated that 1,25(OH)2D3 inhibited podocyte uPAR expression in vitro and in vivo, which may be an unanticipated off target effect of 1,25(OH)2D3 and explain its antiproteinuric effect in the 5/6 nephrectomy rat FSGS model and the LPS mouse model of transient proteinuria.

## Introduction

Proteinuria is a key feature of kidney glomerular dysfunction, and it is a risk factor for both renal and extrarenal diseases [1]. Emerging clinical and animal studies have demonstrated that vitamin D and its analog reduce proteinuria in patients with IgA nephropathy [2], non-dialysed chronic kidney disease stage 4–5 [3], [4], diabetic nephropathy [5], and animal models such as subtotally nephrectomized model [6], diabetic nephropathy model [7], adriamycin-induced nephropathy [8], puromycin-induced nephropathy [9], [10]. However, the mechanisms underlying the antiproteinuric effect of vitamin D remains to be fully elucidated.

The common denominator in a variety of kidney diseases is podocyte dysfunction involving proteinuria [11]. Podocytes and their foot processes comprise the outer layer of the kidney ultrafiltration barrier, which is a complex cellular structure selective ultrafiltration [12], [13]. Typically, most cases of proteinuria are associated with foot process effacement. An emerging concept is that podocyte foot process effacement represents an increased motility of podocytes. [14], [15]. This increased motility of podocytes is best translated into foot process dynamics *in vivo*, in which podocytes remain locally attached to the GBM, whereas altered foot process dynamics lead to foot process effacement and proteinuria[15]–[19]. Recently, urokinase receptor (uPAR) has been shown to orchestrates podocyte motility and has a direct role in regulating podocyte foot process structure and function [16]. uPAR, a glycosylphosphatidylinositol(GPI)-anchored protein, has important roles in stem cell mobilization, tumor invasion and metastasis, wound healing and inflammation [21], [22]. Recently, uPAR[16]–[20] and its soluble form (suPAR)[23]–[28] have been shown to be involved in the pathogenesis of podocyte foot process effacement, proteinuria and focal segmental glomerulosclerosis (FSGS). And thus, proteinuria could be reduced by inhibiting podocyte uPAR expression[17]–[19].

Several studies showed that 1,25(OH)2D3,an active form of vitamin D [29], could down-regulate breast tumor cells invasion via inhibiting uPAR expression[30]–[31]. However, in this study, we showed that 1,25(OH)_2_D_3_ inhibited the expression of podocyte uPAR, a recently confirmed pathogenic factor causing podocyte injury and proteinuria [16]. These findings that 1,25(OH)_2_D_3_ inhibited podocyte uPAR may provide a new insight into the mechanisms underlying its well-known antiproteinuric effect.

## Results

### 1,25(OH)2D3 Treatment Inhibited Proteinuria, Glomerulosclerosis and Podocyte uPAR Induction in 5/6 Nephrectomy (NTX Rats) Rats

**Figure 1:**
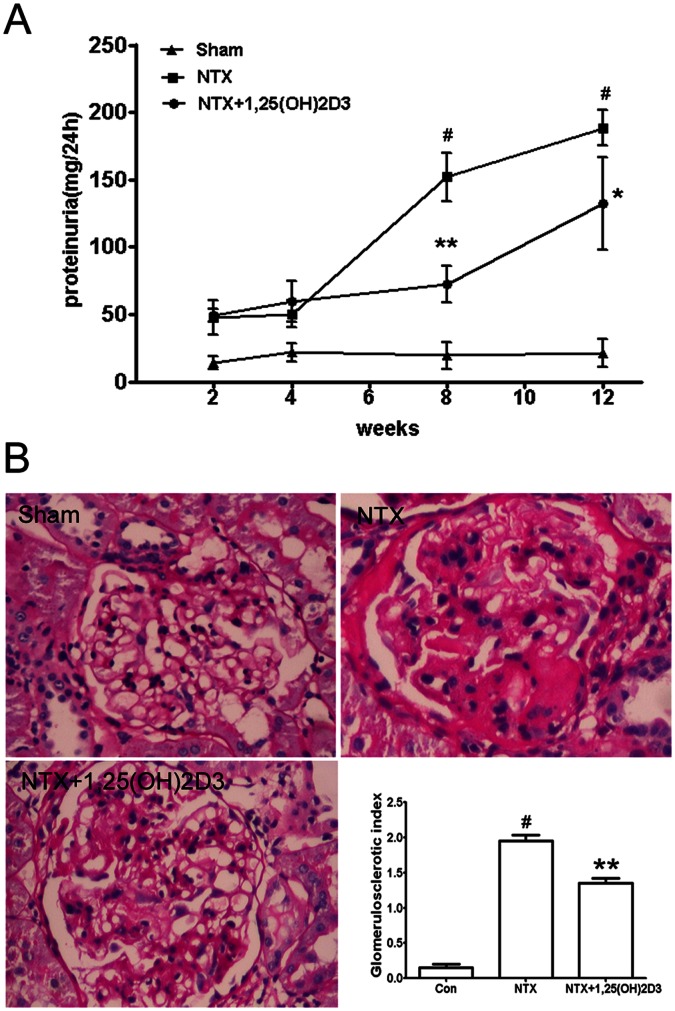
1,25(OH)2D3 ameliorated proteinuria and glomerulosclerosis in 5/6 nephrectomy rats (NTX rats). (**A**) At time points of 8 and 12 weeks, the proteinuria in 1,25(OH)2D3-treated NTX rats were lower than in untreated NTX rats. (**B**) 1,25(OH)2D3 treatment inhibited glomerulosclerosis in NTX rats at the time point of 12 weeks. As expected, NTX rats showed a marked glomerulosclerosis. Treatment of NTX rats with 1,25(OH)2D3 reduced glomerulosclerosis. All values are expressed as means ± SD. *P<0.05 versus untreated NTX rats; **P<0.01 versus untreated NTX rats; #P<0.01 versus sham rats. Periodic Acid-Schiff stain, original magnification×400.

**Figure 2:**
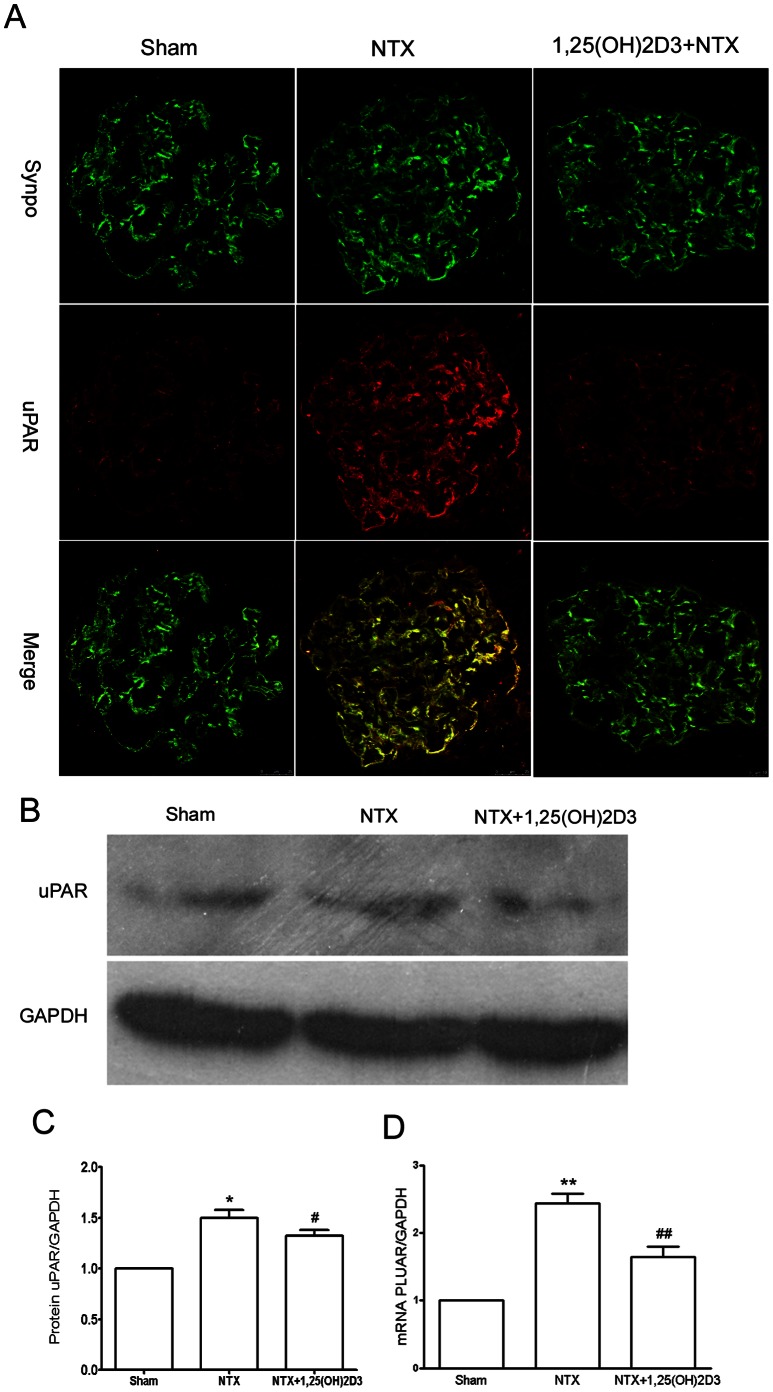
1,25(OH)2D3 inhibited podocyte uPAR induction in NTX rats. (**A**) Double immunofluorescence staining for uPAR (red) and synaptopodin (synpo, green), a podocyte marker, in glomeruli from sham rats or NTX rats treated with vehicle or 1,25(OH)2D3. NTX rats showed an increased expression of uPAR protein in podocytes. 1,25(OH)2D3 substantially inhibits uPAR induction. (**B and C**) Western blot analysis was performed on kidney glomeruli isolated from rats. uPAR protein expression was up-regulated in NTX rats. Treatment with 1,25(OH)2D3 inhibited uPAR protein expression. (**D**) Quantitative real-time RT–PCR was performed on kidney glomeruli isolated from rats. PLAUR mRNA was up-regulated in NTX rats. Treatment with 1,25(OH)2D3 inhibited PLAUR mRNA expression. All values are expressed as means ± SD. *P<0.05, **P<0.01 versus sham rats; #P<0.05, ##P<0.01versus untreated NTX rats.

We tested the anti-proteinuric effect of 1,25(OH)2D3 in 5/6 nephrectomy rats (NTX rats). This model resembled FSGS, a podocyte-related proteinuric kidney disease in human being and is featured nephron loss leading to proteinuria, podocyte dysfunction, glomerulosclerosis, and progressive renal dysfunction [6], [32]. We fed male Sprague-Dawley 5/6 nephrectomy rats once daily with vehicle or 1,25(OH)2D3.and left sham-operated rats with vehicle. Vehicle-treated NTX rats developed heavy proteinuria compared with vehicle-treated sham-operated rats **(** **Figure 1A** **)**. Meanwhile, 1,25(OH)2D3 attenuated proteinuria at time points of 8 weeks and 12 weeks **(** **Figure 1A** **).** (1,25(OH)2D3-treated NTX rats at 8 weeks: 72.41±27.47 mg/24 h versus vehicle-treated NTX rats at 8 weeks: 162.39±27.62 mg/24 h. 1,25(OH)2D3-treated NTX rats at 12 weeks: 131.93±71.04 mg/24 h versus vehicle-treated NTX rats at 12 weeks: 188.31±29.82 mg/24 h, **Figure 1A**). We then wondered whether uPAR expression was elevated in the NTX rats. Morphologically, there was low expression of uPAR in glomeruli from the sham rats **(** **Figure 2A** **)**. uPAR was partially localized in podocytes, as indicated by colabeling with synaptopodin. In contrast, expression of uPAR protein in the NTX rats **(** **Figure 2A** **)** was substantially increased in podocytes. After 1,25(OH)2D3 treatment, we found a substantial reduction of uPAR protein expression in NTX rats **(** **Figure 2A,B and C** **)**. We then performed real-time quantitative PCR with kidney cortex isolated from these rats. We analyzed PLAUR expression in RNA samples from NTX rats. We found low level PLAUR mRNA expression in sham rats. In contrast, NTX rats had a significant increase in PLAUR mRNA expression **(** **Figure 2D** **)**. Notably, we found that, 1,25(OH)2D3 treatment inhibited podocyte uPAR induction in NTX rats.

As NTX rats in the advanced-stage show marked glomerulosclerosis [32], we then sought to examine the effect of 1,25(OH)2D3 on glomerulosclerosis at the time point of 12 weeks. As expected, TNX rats showed significant glomerulosclerosis (**Figure 1B**). Treatment of TNX rats with 1,25(OH)2D3, reduced glomerulosclerosis (**Figure 1B**).

### 1,25(OH)2D3 Inhibited Proteinuria, Effacement of Podocytes Foot Processes and Podocyte uPAR Induction in LPS-induced Proteinuric Mice (LPS Mice)

**Figure 3:**
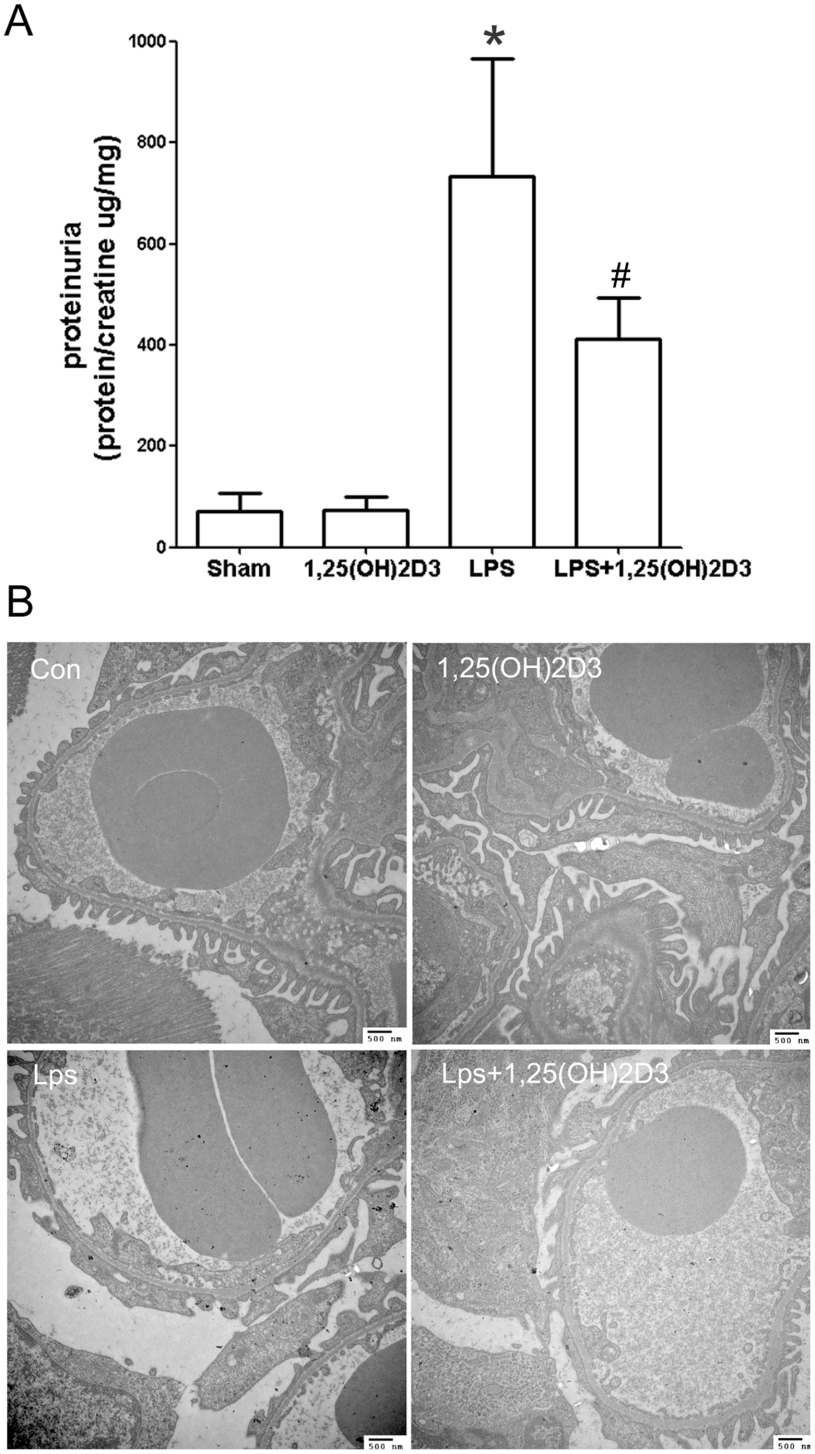
1,25(OH)2D3 ameliorated proteinuria and podocytes foot process effacement in LPS-induced proteinuric mice (LPS mice). (**A**) Compared with LPS mice, 1,25(OH)2D3 significantly reduced proteinuria. (**B**) Compared with control mice, LPS mice showed foot process effacement, a marker of podocyte injury under electronic microscope, and when treated with 1,25(OH)2D3, foot process effacement was alleviated. All values are expressed as means ± SD. *P<0.01, versus control; #P<0.05 versus LPS mice. Transmission electron microscopy original magnification×12000.

We next wondered whether 1,25(OH)2D3 exerts its antiproteinuric effect in LPS-induced proteinuric mice. We fed LPS-injected C57BL/6 mice with either vehicle, or 1,25(OH)2D3, and left control mice with vehicle. Compared with vehicle-treated control mice, vehicle-treated LPS mice developed proteinuria **(** **Figure 3A** **)**. In contrast, proteinuria in 1,25(OH)2D3-treated LPS mice was significantly lower than in vehicle-treated LPS mice (P<0.01; **Figure 3A**).

It is well known that LPS-induced proteinuric mice are featured with changing in altered podocyte foot process dynamics result in foot process effacement and proteinuria[33]–[34]. To explore whether 1,25(OH)2D3 has a role in regulating podocyte foot process structure and function, we observed podocyte foot process structure by transmission electron microscopy. Compared with untreated LPS mice, LPS treated mice shows significant foot process effacement **(** **Figure 3B** **)**. Treatment of LPS treated mice with 1,25(OH)2D3, reduced foot process effacement **(** **Figure 3B** **)**, indicating the anti-proteinuric effect of 1,25(OH)2D3 may be associated with its altering podocyte foot process dynamics and structure action.

**Figure 4:**
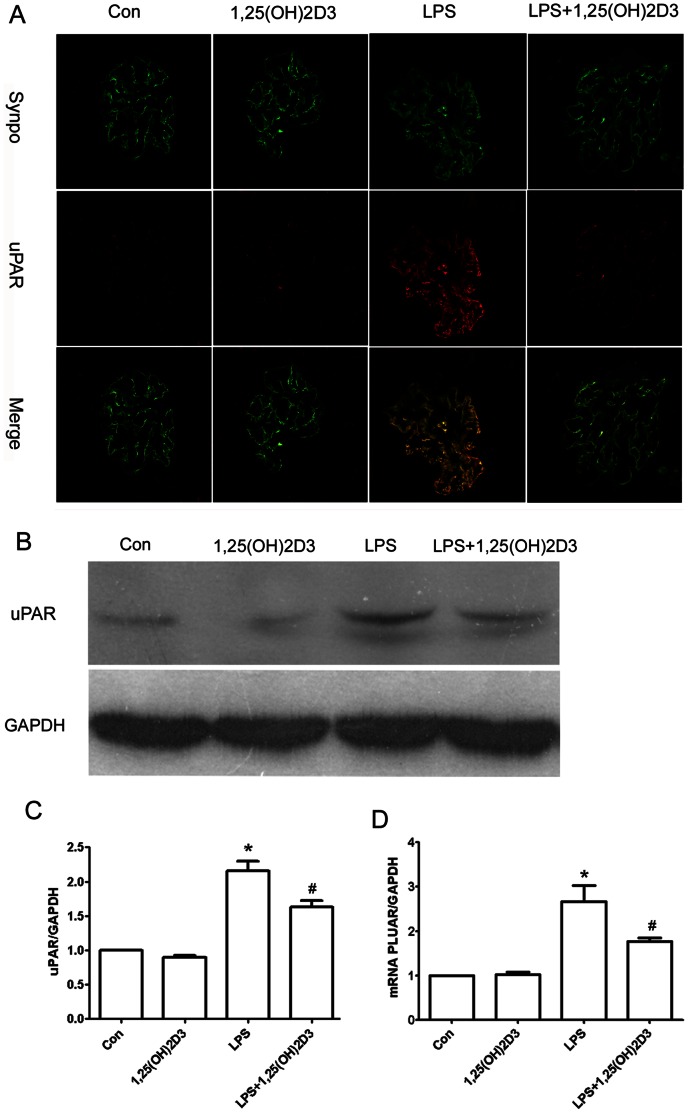
1,25(OH)2D3 inhibited podocyte uPAR induction in LPS mice. (**A**) Double immunofluorescence staining for uPAR (red) and synaptopodin(synpo, green). NTX rats showed an increased expression of uPAR protein in podocytes. 1,25(OH)2D3 substantially inhibited uPAR induction. (**B and C**) Western blot analysis was performed on kidney glomeruli isolated from mice. uPAR protein expression was up-regulated in vehicle-treated LPS mice. However, 1,25(OH)2D3 inhibited uPAR expression. (**D**) Quantitative real-time RT-PCR was performed on kidney glomeruli isolated from mice. PLAUR mRNA was up-regulated in LPS mice and was inhibited by 1,25(OH)2D3 treatment. All values are expressed as means ± SD. *P<0.01, versus control; #P<0.05 versus LPS mice.

We then asked whether uPAR expression was elevated in the LPS mice. Morphologically, there was low expression of uPAR in glomeruli from the control mice **(** **Figure 4A** **)**. uPAR was partially localized in podocytes, as indicated by colabeling with the podocyte marker synaptopodin [31]. In contrast, expression of uPAR protein in the LPS mice **(** **Figure 4A** **)** was substantially increased in podocytes. Interestingly, after 1,25(OH)2D3 treatment, we found a substantial reduction of uPAR protein expression in the LPS mice **(** **Figure 4** **A,B,C)**. We then performed real-time quantitative PCR with kidney cortex isolated from these mice. We analyzed *PLAUR* (encoding uPAR) expression in RNA samples from LPS mice. We found low level *PLAUR* mRNA expression in control mice. In contrast, the LPS mice had a significant increase in *PLAUR* mRNA expression (**Figure 4D**). Of note, we found that 1,25(OH)2D3 inhibited podocyte uPAR induction in LPS-induced proteinuric mice.

### 1,25(OH)2D3 Inhibits uPAR Induction in Podocytes and Podocyte Motility

**Figure 5:**
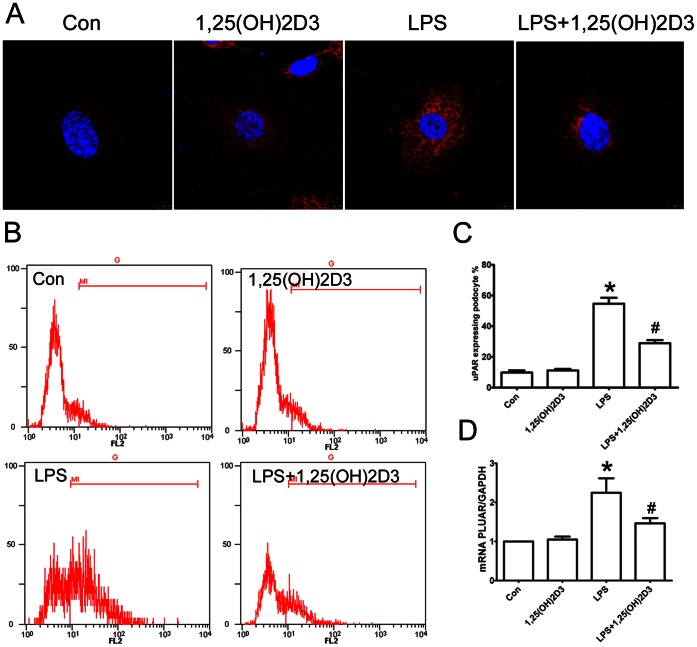
1,25(OH)2D3 inhibited podocyte uPAR induction in vitro. (**A**) Immunofluorescence staining for uPAR protein (red) in cultured differentiated podocytes. (**B and C**)The LPS-treated podocytes showed an increased expression of uPAR protein as indicated by flow cytometry, and, when treated with 1,25(OH)2D3, podocytes showed a significant reduction in uPAR expression. (**D**) Quantitative real-time RT-PCR showed that PLAUR mRNA (encoding uPAR) was up-regulated in LPS-treated podocytes, and was inhibited by treatment of 1,25(OH)2D3. All values are expressed as means ± SD. *P<0.01 versus control; #P<0.01 versus LPS treated podocytes.

To understand whether 1,25(OH)2D3 could inhibit uPAR induction in podocytes, we treated cultured differentiated podocytes [35] with LPS alone (50 mg/L), 1,25(OH)2D3 alone (1 nmol) LPS (50 mg/L) plus 1,25(OH)2D3 (1 nmol). After treatment with LPS, an increased expression of uPAR protein **(** **Figure 5** **A, B and C)** and PLAUR mRNA **(** **Figure 5D** **)** was observed in the podocytes. In contrast, when plus treated with 1,25(OH)2D3, the expression of uPAR protein (Figure 1 A,B and C) and PLAUR mRNA **(** **Figure 5D** **)** was significantly inhibited, indicating that 1,25(OH)2D3 has an inhibitory effect on uPAR expression in podocytes.

**Figure 6:**
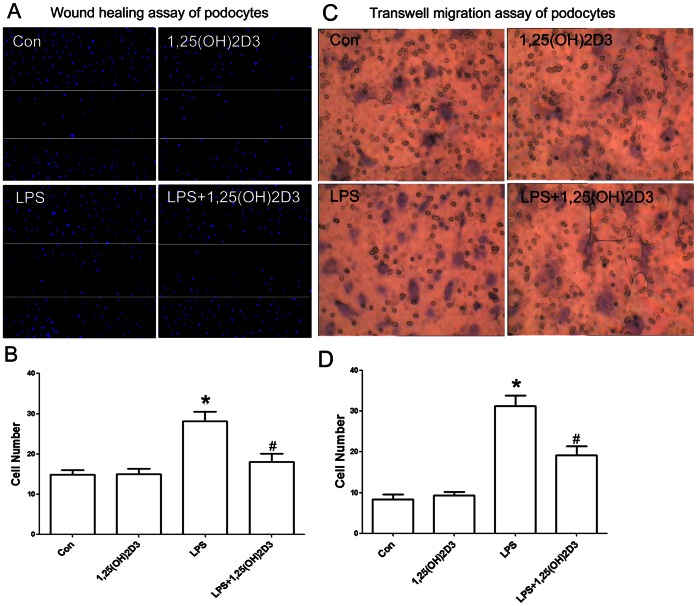
1,25(OH)2D3 inhibited podocyte motility. (**A and B**) Wound healing assay of podocyte grown on vitronectin. LPS treatment for 24 h promoted directed migration of podocytes, and 1,25(OH)2D3 reduced directed migration significantly. (**C and D**) As similar in A and B, when treated with 1,25(OH)2D3, the random motility of podocytes was inhibited significantly. All values are expressed as means ± SD. *P<0.01 versus control; #P<0.01 versus LPS-treated podocytes.

As uPAR is a motility-associated molecule [21], [22] and podocyte motility is regarded as a surrogate indicator for proteinuria and effacement of podocyte foot processes in vivo[16]–[20], we next explore whether 1,25(OH)2D3 has a role in inhibiting cell motility of podocytes in vitro. We first studied podocyte motility before and after 1,25(OH)2D3 treatment using transwell migration assay to assess the random migration of podocytes on vitronectin, a known binding partner of uPAR. LPS treatment for 24 h significantly promoted the migration of podocytes **(** **Figure 6** **A,B)**. In contrast, after plus treatment with 1,25(OH)2D3, the number of migrating podocytes decreased **(** **Figure 6A, B** **)**.

We also analyzed the effect of 1,25(OH)2D3 on the spatial motility of podocytes with a scrape-wound assay. As compared with the control, LPS treatment significantly promoted podocyte wound closure **(** **Figure 6** **C,D)**. In contrast, treatment with 1,25(OH)2D3 reduced podocyte-directed motility **(** **Figure 6C,D** **)**. Together, these data show that1,25(OH)2D3 inhibits podocyte motility as well as podocyte uPAR expression.

## Discussion

Our results demonstrated that 1,25(OH)2D3 inhibited podocyte uPAR mRNA and protein synthesis in vitro and in vivo, which may be an unanticipated effect of 1,25(OH)2D3 and explain its antiproteinuric effect in the 5/6 nephrectomy rat FSGS model and the LPS mouse model of transient proteinuria.

Our findings provided a new insight into the mechanisms for 1,25(OH)2D3’s well known anti-proteinuric effect, which is independent of its regulation of calcium, phosphate metabolism. In this study, 1,25(OH)2D3 ameliorated proteinuria and inhibited podocyte uPAR induction, a pathogenic pathway activated in podocytes during the development of podocyte damage and proteinuria [16]. uPAR is a proteinase receptor and is also involved in nonproteolytic pathways, mainly through interactions with other plasma membrane proteins such as integrins, caveolin and G-protein-coupled receptors [21], [22]. uPAR, together with β3 integrin and vitronectin, mediates podocytes dysfunction and development of proteinuria in mice [16], [17]. It has been reported that uPAR-deficient mice(Plaur−/− mice) were protected from proteinuria in response to LPS and most notably, when uPAR was reconstituted, Plaur−/− mice developed heavy proteinuria after LPS injection [16]. Recently, the soluble form of uPAR has also been identified as the circulating FSGS factor leading to proteinuria [17]. Previous studies have shown that active vitamin D inhibits uPAR expression in breast tumor cells [31]. Here, we provide a new evidence in support of 1,25(OH)2D3’s inhibitory action on uPAR expression not only in LPS-treated podocytes but also in two animal models of proteinuria, suggesting that the antiproteinuric effect of 1,25(OH)2D3 may be related to a podocyte uPAR-related mechanism. In the NTX rats, a model resembling FSGS in human beings [6], [32], our results showed that 1,25(OH)2D3 reduced proteinuria and glomerulosclerosis as well as inhibited podocyte uPAR expression. The expression of podocyte uPAR protein in the NTX rats was substantially increased, when treated with 1,25(OH)2D3, uPAR expression was substantially reduced. We also found that 1,25(OH)2D3 inhibited podocyte uPAR expression and induced remission of proteinuria in LPS-induced proteinuric mice, indicating that the antiproteinuric action of 1,25(OH)2D3 may be partially related to its inhibition of uPAR. However, the two animal models, used in this study, induce proteinuria via different mechanisms; one is due to inflammation and the other one by hemodynamic changes. The results of the current study did not rule out the possibility that the anti-proteinuric effect of 1,25(OH)2D3 is non-specific.

We also noted that LPS-induced proteinuric mice(LPS mice) is a tool to investigate podocyte-specific proteinuria in other studies[33]–[34]. A new concept is that LPS causes proteinuria by targeting podocytes and not other cell types in the kidney, which is in keeping with the observation that podocyte-specific expression of cathepsin L-resistant dynamin [14], [36] or synaptopodin [33] is sufficient to safeguard against LPS-induced proteinuria. Our evidences demonstrated 1,25(OH)2D3 inhibited uPAR induction in podocytes in LPS-induced proteinuric mice, indicating that the antiproteinuric effect of 1,25(OH)2D3 may be directly attributable to its action on podocytes. However, due to the broad effects of LPS on inflammation induction and LPS can cause a variety of immune- and cellular disorders [37], we could not rule out the possibility that the effect of 1,25(OH)2D3 on LPS-induced proteinuria may target to other cells.

uPAR, a molecule associated with cell motility, is highly expressed on cell surface of diseased podocytes [21], [22]. Most cases of proteinuria are associated with effacement of podocyte foot processes, which represents podocyte dynamics in vivo or motility of podocytes [15], [16]. There, podocytes stay attached to the GBM, but altered motility of podocytes results in podocyte foot process effacement and proteinuria. Previous studies have shown that active vitamin D inhibits tumor cells metastases[30]–[31]. To wonder whether 1,25(OH)2D3 inhibits cell motility of podocytes *in vitro*, we examined motility of podocytes when treated with 1,25(OH)2D3. Our data showed that 1,25(OH)2D3 reduced podocyte-directed motility and random migration. To observe the effects of 1,25(OH)2D3 on podocyte motility *in vivo* or podocyte foot processes effacement in LPS mice, ultrastructural analysis were performed by transmission electron microscopy. Our results showed that 1,25(OH)2D3 ameliorated foot process effacement in LPS mice, which may be interpreted that *in vivo* podocyte motility was inhibited by 1,25(OH)2D3.

The mechanisms underlying vitamin D’s inhibition proteinuria remain to be fully elucidated. Recently, experimental data suggested that vitamin D may protect podocytes by targeting multiple pathways, including the renin-angiotensin system, Wnt/β-catenin pathway and pro-apoptotic pathway [29]. And vitamin D has also been shown to reduce proliferating cell nuclear antigen, cyclin-dependent kinase inhibitor p27, desmin (a marker of early podocyte damage), local renin-angiotensin system [6], [7], [29], [38], [39]. The active Vitamin D also prevents podocyte apoptosis by inhibiting p38 MAPK (mitogen-activated protein kinase) and activating the PI3K (phosphatidylinositol 3-kinase)/AKT signaling pathway [9]. Although diabetic vitamin D receptor knockout mice developed more severe proteinuria and glomerulosclerosis due to increased glomerular basement membrane thickening and podocyte effacement [7],the present study could not show that the effect of 1,25(OH)2D3 on uPAR is through the vitamin D receptors and this renoprotective effect might be an off-target effect. Together, our findings provided a new insight into the renoprotective effect of 1,25(OH)2D3, and might offer a potential target in preventing the progression of kidney diseases.

## Methods

### Animals

The animal study has been approved by Research Ethics Committee, Guangdong General Hospital, Guangdong Academy of Medical Sciences (No.GDREC 2010071A). We purchased Sprague-Dawley rats and C57BL/6 mice from Laboratory Animal Center, Sun Yat-sen University, China.

### Animal Models and Treatment with 1,25(OH)2D3

The 5/6 nephrectomy FSGS model(NTX rats) was induced in male Sprague-Dawley rats(Laboratory Animal Center, Sun Yat-sen University, China), initial weight, 250 to 300 g ) by performing surgical resection. One week later, all animals having undergone 5/6 renal mass reduction were then randomized to (1) receive 1,25(OH)2D3 (Roche, Pharmaceuticals) once daily by gastric gavage (0.3 mg kg/day, 1,25(OH)2D3+ group, n = 13); (2) or receive olive oil (equal volume, NTX group, n = 14). The rats of sham-operated group (Sham group, n = 14) also received olive oil once daily by gastric gavage. We collected urine at time points of 2,4, 8 and 12weeks for Bradford protein analysis(Bradford protein assay kit, P0006, Beyotime, China). For each group, 3 rats were sacrificed at 2,4and 8 weeks after randomization, respectively. The remaining rats (4 for NTX+1,25(OH)2D3, 5 for NTX, and 5 for Sham) were sacrificed at 12 weeks.

The extent of glomerulosclerosis was determined in 3 µm kidney sections and a glomerulosclerotic index was then calculated, as described previously [17]. In brief, 60 glomeruli from each rat were examined in a masked protocol. The degree of sclerosis in each glomerulus was graded on a scale of 0–4 as described previously with Grade 0, normal; Grade 1, sclerotic area up to 25% (minimal); Grade 2, sclerotic area 25–50% (moderate); Grade 3, sclerotic area 50–75% (moderate to severe) and Grade 4, sclerotic area 75–100% (severe).

For the LPS mouse model of transient proteinuria (LPS mice) [33]–[34], 32 C57BL/6 male mice were randomly divided into four groups: normal control group(Con), LPS induced group (LPS), 1,25(OH)2D3 treated alone group(1,25(OH)2D3) and LPS plus 1,25(OH)2D3 treated group (LPS+1,25(OH)2D3). We injected C57BL/6 mice intraperitoneally with 300 µg LPS(first day 200 µg, second day 100 µg, L-2880,Sigma-Aldrich, USA). Controls received the same volume of sterile LPS-free saline. For 1,25(OH)2D3 treatment, we gavage mice with 1,25(OH)2D3 2.5 µg/kg.day 2 days before LPS injection and 2 day after LPS injection. For control group and LPS induced group, the mice were gavaged with same volume of Olive oil. We collected the mouse urine for 24 hours after LPS injection. All were executed at the third day.

### Transmission Electron Microscopy (TEM)

For transmission electron microscopy, ultrathin sections (60 to 100 nm) were cut from cortical kidney tissue samples embedded in epon resin, using an ultramicrotome (Leica), collected on copper grids, and stained with uranyl acetate and lead citrate. Ultrathin sections were stained with uranyl acetate for 10 min and subsequently in Reynolds lead citrate for 2 min. Ultrastructural analysis was performed by transmission electron microscopy.

### Cell Culture

Conditionally immortalized mouse podocytes were kindly provided by Dr. FR Danesh. (Baylor Medical Hospital, Houston, USA ) and cultured as reported previously [35], [40]. To propagate podocytes, cells were cultivated on BD BioCoat Collagen I plates (BD Biosciences, USA) at 33°C in the presence of 20 U/ml mouse recombinant IFN-γ(CYT-358, ProSpec-Tany TechnoGene Ltd, Israel ) to enhance expression of a thermosensitive T antigen. To induce differentiation, podocytes were maintained at 37°C without IFN-γ for 10–14 days. To quiescent the cells, differentiated podocytes were serum-starved overnight before experiment. Immortalized mouse podocytes were cultured and randomized into four groups: control group(Con), LPS (50 mg/L) group(LPS), 1,25(OH)2D3(1 nmol) alone group(1,25(OH)2D3), LPS (50 mg/L) plus 1,25(OH)2D3 (1 nmol) group(LPS+1,25(OH)2D3).

### Flow Cytometry

Flow cytometry assay is used to assess uPAR on cell surface of podocytes after 24 h of these treatment, approximately 10^6^/ml cells were trypsinized, washed with PBS(Ca^2+^ free), and incubated with Phycoerythrin (PE)-conjugated rat monoclonal anti-mouse uPAR (FAB531P,R&D Systems, USA ) for 20 minutes at 4°C. Cells were then washed in PBS (Ca^2+^ free) twice. As a control for this analysis, cells in a separate tube were treated with PE-labeled rat IgG_2A_ antibody. Data were collected by Cell Lab Quanta™ SC Flow Cytometry System and analysed using Cell Lab Quanta™ SC analyse (Beckman Coulter, Inc, USA).

### Immunocytochemistry

Murine kidneys and cultured podocytes were harvested and snap-frozen according to standard protocols and fixed after sectioning in ice-cold acetone for 10 minutes. For immunofluorescent labeling, sections were washed once with PBS, permeabilized with 0.5% Triton X-100 in PBS and incubated with blocking solution (5% BSA) for 20 minutes at room temperature before further incubation with one of the primary antibodies (synaptopodin (N-14), sc-21536; uPAR(FL-290), sc-10815) for 2 hours at room temperature. For double labeling, sections were washed three times with PBS for 5 minutes, and one of the secondary antibodies (Alexa Fluor® 488 monkey anti-goat IgG (H+L), A11055; Alexa Fluor® 635 goat anti-mouse IgG (H+L), A31575; Alexa Fluor® 546 goat anti-rabbit IgG (H+L), A11010, Invitrogen, USA; rabbit anti-goat IgG-FITC, sc-2777, Santa Cruz Biotech, USA; NorthernLights™ anti-mouse IgG-NL637,NL008, R&D Systems, USA) was applied for 2 hours. Pictures were captured with confocal microscopy (Leica Microsystems). All images were analyzed by two investigators blinded to the identity of the samples.

### Real-time Quantitative PCR

Real-time quantitative PCR for cultured podocytes and kidney glomeruli isolated from rodent proteinuric models was performed as recommended in PrimeScriptTM RT reageny Kit (Takara Bio Inc, Japan)using the following primers:rat Plaur (NM_017350;mouse Plaur (NM_011113.3);rat GAPDH(NM_017008);mouse GAPDH(NM_001001303); rat Plaur (forward, AGATGTGCTGGGAAACCG; reverse, CAGGGAGGCAATGAGGAT ) yielding a 196-bp product; mouse Plaur (forward, AAGCCTGCAATGCCGCTATC; reverse, GGGTGTAGTTGCAACACTTCAGGA) yielding a 182-bp product.

### Transwell Migration Assay

Transwell cell culture inserts (pore size 5 µm; Costar Corporation, USA) were coated with vitronectin, rinsed once with DPBS and placed in DMEM medium (10%FBS) in the lower compartment. For each experiment, 1×10^4^·ml^−1^cultured differentiated podocytes were seeded in the inserts and allowed to migrate for 24 h while being incubated at 37°C. Non-migratory cells were removed from the upper surface of the membrane, and migrated cells were fixed with 4% paraformaldehyde and stained with Crystal Violet Solution (Sigma-Aldrich, USA). The number of migrated cells was counted using phasecontrast microscopy with a ×20 objective on an microscope (Olympus) in the centre of a membrane (one field). The data presented represent the mean±sd of three independent experiments.

### Wound Healing Assay

Cultured differentiated podocytes (each 1×10^5^ ml^−1^) were seeded overnight on vitronectin-coated coverslips in six-well plates. Each coverslip was then scratched with a sterile 200 µl pipette tip, washed with PBS and placed into fresh medium. After 24 h, cells were fixed with cold methanol, permeabilized with 0.5% Triton X-100 in PBS and cell nuclei were stained with DAPI (Roche Diagnostics). Pictures were captured by phase-contrast microscopy under a ×10 objective on an microscope (Leica Microsystems) at 0 and 24 h after scratching, and the number of cells that had migrated into the same-sized square fields were counted. The data presented represent the mean±sd of five independent experiments.

### Western Blot Analysis

Protein expression of uPAR were determined by Western blot analysis. Briefly, kidney cortex isolated from rodent was homogenized in 1 ml of tissue lysis buffer. Samples were centrifuged at 3,000 g for 15 min, and the supernatants were assayed. After being mixed with SDS-PAGE (NuPAGE) sample buffer and boiled for 5 min, samples were electrophoresed on 10% SDS polyacrylamide gels and transferred to PVDF membranes (Millipore) for 2 h at 40 V. Membranes were blocked for 30 min with Tris-buffered saline that contained 5% BSA (5% BSA/TBS) and incubated with diluted primary antibody including anti-uPAR(FL-290), sc-10815 (1∶1000, Santa Cruz Biotechnology), anti-GAPDH (1∶1000, Santa Cruz Biotechnology) overnight at room temperature in 5% BSA/TBS that contained 0.05% Tween 20. The membranes were washed and developed using the enhanced chemiluminescence system (Applygen Technology).

### Statistical Analysis

We assessed statistical significance by one-way ANOVA analysis of variance followed by LSD test for comparison between two groups. P<0.05 was considered significant. All values are expressed as mean±s.d.
